# Access to haemodynamic evaluation tools in middle-income countries: a survey of 1593 anaesthetists and intensivists from 39 nations

**DOI:** 10.1016/j.bjao.2025.100515

**Published:** 2025-12-15

**Authors:** Frederic Michard, Jigeeshu Divatia, Flavio E. Nacul, Syarifah N.N.S. Masri, Suraphong Lorsomradee, Vanina Kanoore-Edul, Eduardo Kattan, Asli Z. Demir, Francisco Chacon-Lozsan, Ever L. Rojas-Diaz, Manu L.N.G. Malbrain, Michelle S. Chew

**Affiliations:** 1 MiCo, Vallamand, Switzerland; 2 Department of Critical Care Medicine, Lilavati Hospital and Research Centre, Mumbai, India; 3 Critical Care Medicine, University Hospital, Federal University of Rio de Janeiro & Pro-Cardiaco Hospital, Rio de Janeiro-RJ, Brazil; 4 Department of Anaesthesiology and Intensive Care, Hospital Canselor Tuanku Muhriz, Universiti Kebangsaan Malaysia, Kuala Lumpur, Malaysia; 5 Department of Anesthesiology, Faculty of Medicine, Chiang Mai University, Chiang Mai, Thailand; 6 División de Terapia Intensiva, Hospital Juan A. Fernández, Ciudad Autónoma de Buenos Aires, Argentina; 7 Departamento de Medicina Intensiva, Facultad de Medicina, Pontificia Universidad Católica de Chile, Santiago, Chile; 8 Department of Anesthesiology, Bilkent City Hospital, Turkey University of Health Sciences, Ankara, Turkey; 9 Anesthesia and Intensive care Unit, Péterfy Sándor Hospital, Budapest, Hungary; 10 Department of Intensive and Critical Care Medicine, Academic Hospital Fundación Santa Fe de Bogota, Bogotá, Colombia; 11 First Department of Anaesthesiology and Intensive Therapy, Medical University of Lublin, Lublin, Poland; 12 Department of Perioperative Medicine and Intensive Care, Karolinska University Hospital, Huddinge, Sweden

**Keywords:** anaesthesiology, echocardiography, haemodynamic monitoring, inequity, intensive care, perioperative medicine, techquity

## Abstract

**Background:**

Approximately 75% of the world’s population lives in middle-income countries (MICs), where access to haemodynamic evaluation tools may be limited, exacerbating global healthcare disparities.

**Methods:**

We conducted an online survey of anaesthetists and intensivists working in MICs, inviting them to complete 15 questions on bedside haemodynamic evaluations and access to haemodynamic monitoring tools.

**Results:**

We analysed 1593 valid questionnaires from 20 Upper and 19 Lower MICs. Most respondents (66%) worked in academic hospitals, 43% in private hospitals, and 20% in non-academic public hospitals. Respondents worked in ICUs (39%), operating rooms (38%), or both (23%). Nearly all had access to central venous catheters (99%) and invasive radial arterial pressure monitoring (91%). Fewer than two-thirds (63%) reported access to echocardiography, and only 37% had access to cardiac output monitoring systems when needed. The main barriers were the cost of monitors (54%) and the cost of disposable sensors (52%). Notably, 72% indicated they would use cardiac output monitoring equipment more frequently if costs were reduced. Most respondents (89%) reported a routine practice of predicting fluid responsiveness before giving a fluid bolus, most commonly with pulse pressure variation (64%) or ultrasound indices (55%). Tissue perfusion was mainly assessed by clinical evaluation (86%), blood lactate (81%), and capillary refill time (63%).

**Conclusions:**

In MICs, less than two-thirds of anaesthetists and intensivists reported having access to echocardiography for haemodynamic assessment. Fewer than 40% have access to cardiac output monitoring systems, mainly attributable to economic constraints. As this report represents a potential concerning equity gap in global healthcare, efforts should be made to prioritise and facilitate access to haemodynamic evaluation tools in MICs.

Haemodynamic instability is frequent during and after high-risk surgery, and in critically ill patients. During surgery, it is primarily driven by co-morbidities of the patient together with the effects of anaesthetics agents on vascular tone and by surgical blood loss. After surgery, and in medical patients, haemodynamic instability may result from multiple factors, including dehydration, bleeding, sepsis, and cardiorespiratory events such as myocardial infarction or pulmonary embolism. If sustained, haemodynamic instability may cause tissue hypoperfusion and hypoxia, potentially progressing to organ failure and death. In resource-limited settings, circulatory failure is the leading cause of mortality after abdominal surgery.^1^

The primary haemodynamic evaluation tools include central venous catheters for measuring central venous pressure (CVP) and oxygenation (ScvO_2_), arterial catheters for continuous blood pressure and pulse pressure variation (PPV) monitoring, cardiac output monitoring systems, near-infrared spectroscopic (NIRS) sensors for estimating end organ oxygenation, and ultrasound devices for point-of-care assessment of cardiac anatomy and function. Haemodynamic evaluationtools help identify specific haemodynamic profiles or phenotypes (e.g. hypovolemic, distributive, or cardiogenic shock) and support timely targeted treatment of haemodynamic instability based on its underlying mechanisms.^2^^,^^3^ Their use is recommended in high-risk surgical^4^^,^^5^ and critically ill^6^ patients and has been associated with improved patient outcomes.^7^, ^8^, ^9^, ^10^ However, surveys and observational studies conducted in high-income countries suggest that these tools, particularly cardiac output monitoring systems, are often underutilised.^11^, ^12^, ^13^, ^14^ Notably, one of the most frequently cited barriers to wider clinical adoption is the cost of equipment.^12^ Approximately 75% of the world’s population lives in middle-income countries (MICs), where access to haemodynamic evaluation tools may be even more limited, further widening global healthcare disparities.

Therefore, we developed a survey to describe the availability and potential barriers to the use of haemodynamic evaluation tools among anaesthetists and intensivists working in MICs.

## Methods

A web-based survey was developed, conducted, and reported in accordance with CHERRIES (Checklist for Reporting Results of Internet ESurveys) guidelines^15^ (see Supplementary material 1). Participation in the survey was voluntary and anonymous, and no patient data were collected. The questionnaire was endorsed by the International Fluid Academy (IFA) and it solicited permission to use responses for analysis and publication.

An anonymous English questionnaire containing 15 short questions was developed using Google Forms (see Questionnaire in Supplementary material 2). FM created the questionnaire draft, which was reviewed, edited, and tested by VKE, FCL, EK, and MSC before being distributed.

The authors and the IFA shared the survey link with anaesthetists and intensivists working in MICs *via* e-mail and social media. A list of 46 pre-selected MICs (Question 1) was sourced from World Bank data (worldbank.com). In 2024, according to the World Bank, MICs are nations with a Gross National Income (GNI) per capita ranging from 1136 to 13 845 USD**.** They are divided into two subcategories**:** Lower MICs, with GNI per capita between 1136 and 4465 USD**,** and Upper MICs, with GNI per capita between 4466 and 13 845 USD**.** For the sake of comparison, the GNI per capita is >50 000 USD in most Western European countries and >80 000 USD in the USA and Switzerland.

No incentive was offered for participation in the survey. Cookies were not used, and an IP check was not possible, preventing the identification of respondents. Reminders were sent after 3 and 6 weeks to maximise the number of respondents, and the database was locked after 2 months.

Questionnaires not filled by an anaesthetist or intensivist (board-certified or resident) working in one of the 46 pre-selected MICs, or with more than three unanswered questions, were considered invalid. When respondents did not permit us to use their responses for publication (Question 15), questionnaires were excluded from the analysis.

Data are presented as numbers, ratios, and percentages. Multiple answers were allowed for several questions (see the Questionnaire in Supplementary material 2), so that the cumulative percentages given in the text or figures may exceed 100%. Because respondents could leave up to three questions unanswered, all reported proportions are relative to the number of responses to each question. Access to haemodynamic tools was compared between respondents working mainly in the operating room (OR) and in the ICU, and between those working in Lower and Upper MICs, using a χ^2^ test. To reduce the chance of false positive findings, we set the statistical significance level at *P*<0.01.

## Results

**Fig 1:**
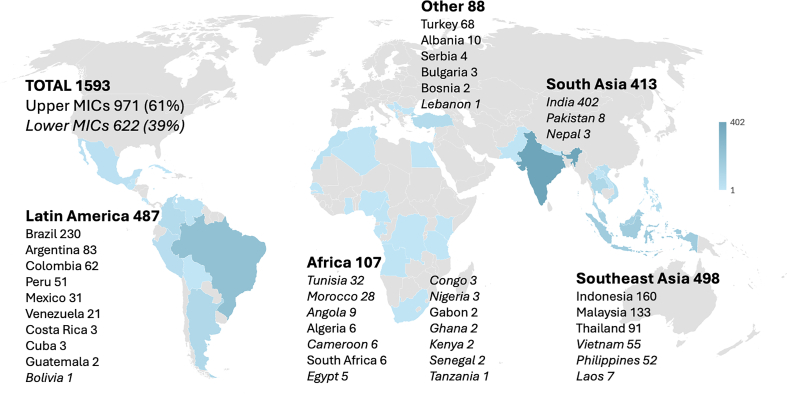
Number of valid questionnaires per region and per middle-income country (MIC). Lower MICs in *italic.*

The survey link was shared from 17 February 2025, and the survey database was locked for analysis on April 17 after receiving 1616 valid questionnaires. Twenty-three respondents did not permit us to use their answers for analysis and publication. Therefore, 1593 questionnaires were available for analysis. Respondents were from 39 different MICs, and the majority (971/1593, 61%) worked in Upper MICs (Fig. 1). Most respondents (1043/1584, 66%) worked in academic hospitals, 43% (679/1584) in private hospitals, and 20% (322/1584) in non-academic public hospitals. Thirty-nine percent (598/1593) worked mainly in the ICU, 38% (626/1593) mainly in the OR, and 23% (369/1593) worked in both environments.

Almost all respondents reported having access to central venous catheters (1569/1590, 99%). Most used it to monitor CVP (1085/1569, 69%) and 50% (777/1569) to measure ScvO_2_. Ninety-one percent of respondents (1457/1593) reported monitoring invasive radial arterial pressure when needed, and 68% (997/1457) did so with a dedicated arterial catheter (*vs* a peripheral venous catheter).

**Fig 2:**
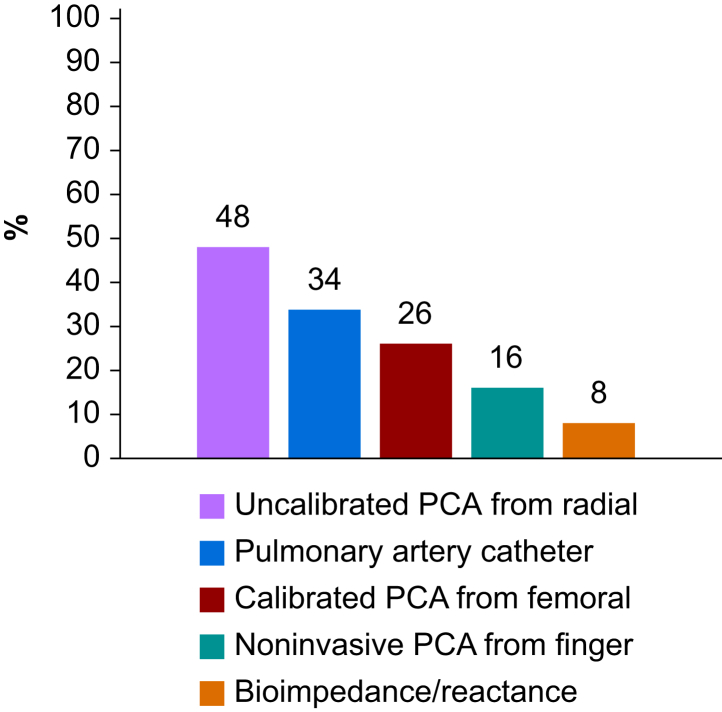
Cardiac output monitoring tools used in middle-income countries. PCA, pulse contour algorithm.

**Fig 3:**
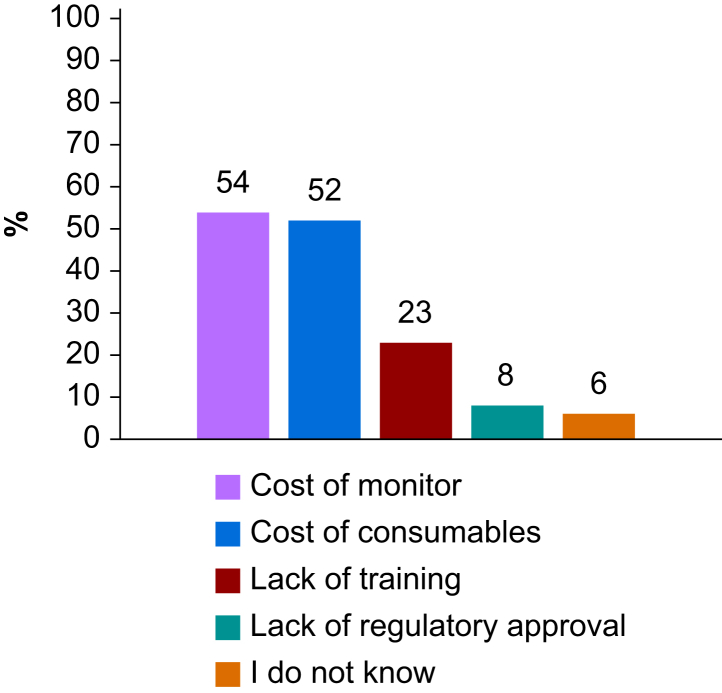
Limitations to accessing cardiac output monitoring tools in middle-income countries.

Less than two-thirds of respondents (1001/1589, 63%) had access to point-of-care echocardiography, and 91% (1443/1588) used cart-based ultrasound devices. Only 14% (226/1588) had access to pocket ultrasound devices. Thirty-seven percent of respondents (590/1581) had access to cardiac output monitoring systems whenever they deemed it necessary. Techniques reportedly used to monitor cardiac output are presented in Figure 2, and reasons given for the limited access to cardiac output monitoring are shown in Figure 3. If cardiac output monitoring tools were less expensive, 72% (1119/1561) of respondents indicated they would use them more frequently, 16% (245/1561) would use them as often as they do today, and 10% (160/1561) would not use them.

**Fig 4:**
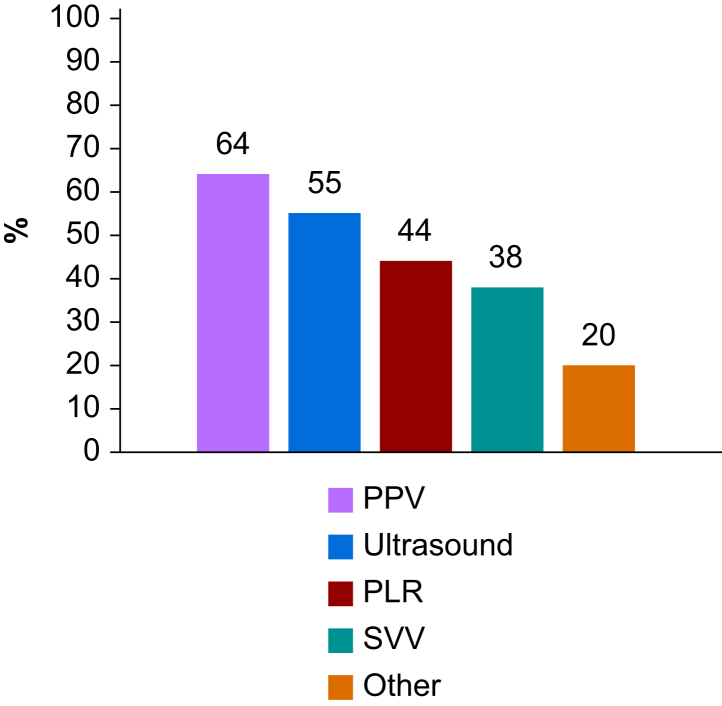
Variables or methods used to predict fluid responsiveness in middle-income countries. PLR, passive leg raising manoeuvre; PPV, pulse pressure variation; SVV, stroke volume variation.

**Fig 5:**
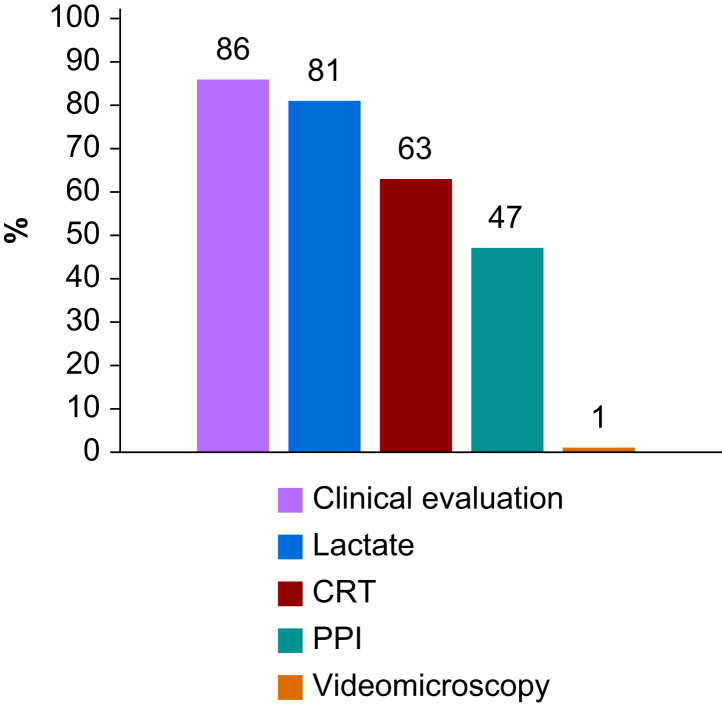
Methods used to assess tissue perfusion in middle-income countries. CRT, capillary refill time; PPI, peripheral perfusion index.

Most respondents (1411/1588, 89%) predicted fluid responsiveness before administering a fluid bolus, and most did so with PPV or ultrasound indices (Fig. 4). To assess tissue perfusion, most respondents relied on clinical evaluation, blood lactate, and the capillary refill time (CRT) (Fig. 5). To assess tissue oxygenation, only 32% (502/1586) of respondents had access to NIRS sensors.

Regarding access to haemodynamic evaluation tools, comparisons between respondents working mainly in the OR and in the ICU and between those working in Lower and Upper MICs are presented in Table 1.

**Table 1:** Proportion of respondents who have access to haemodynamic evaluation tools when needed. Comparison between upper and lower middle-income countries (MICs) and between respondents working mainly in the operating room (OR) and the ICU. NIRS, near-infrared spectroscopy. ∗*P*<0.01 Upper MIC *vs* Lower MIC or OR *vs* ICU.

Access to	Upper MIC	Lower MIC	OR	ICU
	(*n*=971)	(*n*=622)	(*n*=626)	(*n*=598)
Central venous catheterisation	961/969 99%	611/621 98%	611/625 98%	595/597 100%
Radial arterial catheterisation	907/965 94%	534/612 87%∗	555/626 89%	570/598 95%∗
Echocardiography	615/971 63%	386/618 62%	298/624 48%	468/596 79%∗
Cardiac output monitoring	378/971 39%	212/622 34%	229/626 37%	249/598 42%
NIRS sensors	369/970 38%	133/616 22%∗	244/623 39%	135/595 23%∗

## Discussion

This survey focused on access to haemodynamic evaluation tools for surgical and ICU patients in MICs. The findings reveal that access to essential equipment, such as echocardiographic devices and cardiac output monitoring systems, remains a major challenge in MICs. This represents a concerning equity gap in global healthcare.

Although most respondents (91%) reported being able to monitor invasive radial pressure when needed, only 69% did so using a dedicated arterial catheter. The length, diameter, and material of intravascular catheters are known to affect damping phenomena and, consequently, the arterial pressure waveform.^16^^,^^17^ Using a short peripheral venous catheter for arterial pressure monitoring is therefore unlikely to be optimal.^18^ Studies are warranted to assess the impact of this practice on the accuracy of arterial pressure measurements and on cardiac output estimations derived from pulse contour algorithms.

Less than two-thirds of respondents (63%) reported having access to point-of-care ultrasound devices for echocardiography. This is concerning, as echocardiography is widely regarded as the first-line tool for haemodynamic assessment, particularly in ICU patients.^6^^,^^19^ This finding is also somewhat surprising, considering the deployment of smaller and more affordable ultrasound systems over the past few decades.^20^, ^21^, ^22^ It may reflect a lag between the market introduction of low-cost pocket ultrasound devices and their clinical adoption. Limited training and expertise among anaesthesiologists and intensivists in MICs may also be a contributing factor. Additionally, transthoracic ultrasound is often not feasible during surgery, and more than one-third of respondents worked primarily in the OR.

One of the key findings of our survey is that most respondents (63%) lacked access to cardiac output monitoring tools, primarily because of financial constraints. The cost of such monitoring includes both the fixed expense of the monitor and the recurring costs of consumable sensors, such as specialised pressure transducers for pulse contour techniques or thermistor-tipped pulmonary or femoral artery catheters for thermodilution methods.^23^ These two cost components were cited as the main barriers to cardiac output monitoring by 54% and 52% of respondents, respectively, whereas only 23% mentioned lack of training as the main obstacle. Notably, the high expense of cardiac output monitoring systems is also a well-recognised obstacle to their adoption in high-income countries.^12^^,^^23^ We received responses from 39 different MICs that are not comparable in terms of GNI, reimbursement models, medical insurance policies, access to care, and cost of medical equipment. Nevertheless, cost-reducing solutions are likely essential to improve access.

Interestingly, 89% of respondents reported attempting to predict fluid responsiveness before administering a fluid bolus. This is encouraging, as predicting fluid responsiveness is recommended for rational fluid management in both surgical and ICU patients, including those in MICs.^24^, ^25^, ^26^ These results suggest that the educational efforts made over the past 25 yr to promote individualised fluid management are having a positive impact. Almost two-thirds of respondents reported using PPV to predict fluid responsiveness. Although PPV has well-known limitations,^27^ it offers the advantage of being automatically calculated and displayed on most standard multiparameter bedside monitors, making it easier to use than other indices or manoeuvres. In addition, several clinical studies conducted in MICs have reported outcome benefits with PPV-guided strategies.^28^^,^^29^

To assess tissue perfusion, most respondents reported relying on clinical examination, blood lactate levels, and CRT (Fig. 5). The use of CRT has gained popularity in recent years, largely owing to the ANDROMEDA-SHOCK trial, which suggested it could help rationalise haemodynamic management and improve patient outcomes.^30^^,^^31^ Among the available methods for evaluating tissue perfusion, CRT stands out for its simplicity and negligible cost. The peripheral perfusion index, measured *via* pulse oximeters, shares these advantages and offers the benefit of continuous monitoring.^32^^,^^33^ Notably, a substantial proportion of respondents reported using it (Fig. 5). In contrast, only a minority had access to NIRS sensors for somatic or cerebral tissue oxygenation monitoring. This may be explained by the need for a dedicated monitor, costly disposable skin sensors, and controversies regarding their clinical utility.^34^^,^^35^

Several strategies can be envisioned to enhance access to cardiac output monitoring and echocardiography. Some pulse contour techniques can analyse arterial pressure waveforms obtained from standard, low-cost pressure transducers, thereby eliminating the need for specialised transducers that can cost several hundred dollars per patient.^23^^,^^36^ However, these systems still require a dedicated haemodynamic monitor. Integrating pulse contour algorithms directly into standard multiparameter bedside monitors could remove the need for standalone devices and consumables altogether.^37^ Such innovations have the potential to expand the accessibility of cardiac output monitoring while also reducing associated environmental impacts.^23^^,^^37^ The ongoing miniaturisation of ultrasound devices may further democratise their use, although the lack of adequate training remains a significant barrier to widespread adoption. Recent developments have introduced artificial intelligence-powered tools that assist with image acquisition and automate echocardiographic measurements.^38^^,^^39^ They enable less experienced operators to measure haemodynamic variables with good reproducibility and have even been proposed as ‘self-education’ tools.^39^^,^^40^ Consequently, these technological advances could be valuable in resource-limited settings, where both skilled personnel and formal training programmes are often scarce.

Our survey has several limitations. First, because it was hosted on a Google platform, it was inaccessible from China, preventing the inclusion of Chinese respondents. Furthermore, it was in English, which further limits accessibility. Second, participants were not randomly selected and were primarily from academic centres. As such, this potential selection bias in our survey may not reflect the realities faced by clinicians working in small, remote, and non-academic institutions, where access to haemodynamic tools might be even more restricted. Finally, in addition to targeted e-mails, we disseminated the survey *via* social media (primarily LinkedIn), encouraging participants to share the link. This approach prevented us from determining the total number of clinicians who received the survey and, therefore, the response rate.

Our survey also has notable strengths. First, we gathered feedback from more than 1500 anaesthetists and intensivists across 39 different MICs. Second, although Africa was somewhat underrepresented, the other major regions—South America, South Asia, and Southeast Asia—each accounted for roughly one-third of the respondents. Finally, the respondents were well balanced in terms of primary clinical activity, with similar proportions working mainly in the OR (38%) and in the ICU (39%), and about one quarter dividing their time equally between both settings.

### Conclusions

In MICs, fewer than two-thirds of anaesthetists and intensivists have access to echocardiography for haemodynamic assessment, and fewer than 40% can use cardiac output monitoring systems, primarily because of financial constraints. This limited access perpetuates disparities in perioperative and critical care. Prioritising the development and dissemination of affordable haemodynamic evaluation tools is essential to bridging this gap.

## Authors’ contributions

Study conception and manuscript draft: FM

Questionnaire design and testing: FM, VKE, EK, FCL, MSC

Revising paper: all authors

Approved the final version of the paper: all authors

## Declarations of interest

FM is the managing director of MiCo, a Swiss consulting and research company. MiCo does not sell any medical devices. JD has received lecture fees from Edwards Lifesciences, paid to his former institution, Tata Memorial Hospital in Mumbai. SNNSM has received grants and honoraria from Edwards Lifesciences. MLNGM is co-founder and president of the IFA and the chief medical officer of Medaman. He was member of the medical advisory Board of Pulsion Medical Systems and Baxter. He consults for Becton Dickinson and Medtronic. MSC is the current chair of the Cardiodynamics section of the European Society of Intensive Care Medicine, is an editor for the *British Journal of Anaesthesia*, and has received honoraria from Edwards Lifesciences and Philips Healthcare. The other authors declare that they have no conflicts of interest.
