# Supplementary material for: Access to haemodynamic evaluation tools in middle-income countries: a survey of 1593 anaesthetists and intensivists from 39 nations
Source: BJA Open. 2025 Dec 15;17:100515. doi: 10.1016/j.bjao.2025.100515 (PMC12768935; doi:10.1016/j.bjao.2025.100515)
Supplement: Multimedia component 1 [file mmc1.docx]

Supplementary material S1

CHERRIES checklist

| Design | Web-based survey targeting anesthetists and intensivists working in middle-income countries  Fifteen single- and multiple-choice questions on bedside hemodynamic evaluations and access to hemodynamic tools |
| --- | --- |
| Informed consent and data protection | Participation in the survey was voluntary and anonymous  No patient data was collected  No personal information was collected  Responses were collected and stored on a password protected computer  Question #15 solicited permission to use responses for analysis and publication |
| Development and pre-testing | The questionnaire was developed using Google Forms.  FM created the questionnaire draft, which was reviewed, edited, and tested by VKE, FCL, EK, and MSC before being distributed |
| Recruitment process | Open survey  The authors and the International Fluid Academy shared the survey link with anesthetists and intensivists working in MICs via email and social media  No incentive was offered for participation in the survey |
| Survey administration | The questionnaire was available as a Google Forms link  The survey link was shared from February 17, 2025, and the survey database was locked for analysis on April 17, 2025 |
| Response rate | The survey was disseminated via social media (primarily LinkedIn) and participants were encouraged to share the link. This approach prevented us from determining the total number of clinicians who received the survey and, therefore, the response rate |
| Preventing multiple entries from the same individual | Cookies were not used and IP check was not possible |
| Analysis | Questionnaires not filled by an anesthetist or intensivist (board-certified or resident) working in one of the 46 pre-selected MICs were considered invalid  Questionnaires with more than 3 unanswered questions were considered invalid  When respondents did not permit us to use their responses for publication (Question #15), questionnaires were excluded from the analysis |
