# Supplementary material for: Access to haemodynamic evaluation tools in middle-income countries: a survey of 1593 anaesthetists and intensivists from 39 nations
Source: BJA Open. 2025 Dec 15;17:100515. doi: 10.1016/j.bjao.2025.100515 (PMC12768935; doi:10.1016/j.bjao.2025.100515)
Supplement: Multimedia component 2 [file mmc2.docx]

**Supplementary material S2: QUESTIONNAIRE**

**Hemodynamic evaluations in surgical and critically ill patients from MIDDLE-INCOME countries**

**THANKS** for taking **3 MINUTES** of your time to answer the following questions.

You have to be an **ANESTHETIST**or an**INTENSIVIST** **(residents included)** practicing in a **MIDDLE-INCOME** country (list in the 1st Question) to take this survey.

**1. In which MIDDLE-INCOME country do you work?**

- Albania
- Algeria
- Angola
- Argentina
- Armenia
- Azerbaijan
- Bolivia
- Bosnia & Herzegovina
- Brazil
- Bulgaria
- Cameroon
- Colombia
- Congo
- Costa Rica
- Cuba
- Egypt
- Gabon
- Georgia
- Ghana
- Guatemala
- India
- Indonesia
- Jordan
- Kenya
- Laos
- Lebanon
- Malaysia
- Mexico
- Moldova
- Montenegro
- Morocco
- Nepal
- Nigeria
- Pakistan
- Peru
- Philippines
- Senegal
- Serbia
- South Africa
- Tanzania
- Thailand
- Tunisia
- Turkey
- Ukraine
- Venezuela
- Vietnam

**2. Please confirm you are an ANESTHETIST or INTENSIVIST (residents included)**

- Yes I am, and I work MAINLY in the operating room (OR) for NON-cardiac surgery
- Yes I am, and I work MAINLY in the OR for CARDIAC surgery
- Yes I am, and I work MAINLY in the Intensive Care Unit (ICU)
- Yes I am, and I work equally in the OR and the ICU
- No, I am not and should NOT take the survey
- Other :

**3. MULTIPLE ANSWERS POSSIBLE: Where do you PRACTICE?**

- Academic hospital
- Non-academic public hospital
- Private hospital
- Other :

**4. When needed, can you monitor RADIAL ARTERIAL PRESSURE continuously?**

- No, this is not possible where I work
- Yes, this is possible and I use a dedicated/specific arterial catheter to do so
- Yes, this is possible and I use a short venous catheter to do so
- Other :

**5. MULTIPLE ANSWERS POSSIBLE: When needed, can you insert/use a CENTRAL VENOUS CATHETER?**

- No, this is not possible where I work
- Yes, this is possible and I use it to administer fluids and drugs
- Yes, this is possible, and I may also use it to monitor central venous pressure (CVP)
- Yes, this is possible, and I may also use it to measure central venous oxygen saturation (ScvO2)
- Yes, this is possible, and I may also use it to measure the PCO2 gap
- Other :

**6. Do you have access to CARDIAC OUTPUT monitoring tools (pulmonary artery catheters or pulse contour techniques or bioimpedance/bioreactance devices)?**

- Yes, I have access to cardiac output monitoring tools and use them when/if needed
- Yes, I have access to cardiac output monitoring tools, but cannot use them as often as needed
- Yes, I have access to cardiac output monitoring tools, but I don't use them because of lack of training/expertise
- No, cardiac output monitoring tools are not available where I work
- Other :

**7. MULTIPLE ANSWERS POSSIBLE: Which CARDIAC OUTPUT monitoring tools do you have access to?**

- Pulmonary artery catheter
- Calibrated pulse contour technique working with a femoral catheter
- Minimally invasive uncalibrated/autocalibrated pulse contour technique working with a radial catheter
- Noninvasive uncalibrated/autocalibrated pulse contour technique working with a finger cuff
- Bioimpedance/bioreactance device
- None
- Other

**8. MULTIPLE ANSWERS POSSIBLE: If your access to CARDIAC OUTPUT monitoring tools is LIMITED, what is/are the MAIN REASONS?**

- Cost of cardiac output MONITOR (capital cost)
- Cost of disposable SENSOR (cost per patient)
- Device/technique not approved/available in my country
- Lack of training/expertise
- I don't know
- Not applicable (in case you have access to cardiac output monitoring tools as often as needed)
- Other :

**9. If CARDIAC OUTPUT monitoring tools were LESS expensive**

- I would use them more often
- I would use them as often as today
- I would not use them, cost is not the reason why I don't use them
- Other :

**10. Do you have access to ULTRASOUND techniques to perform point-of-care ECHOCARDIOGRAPHIC evaluations?**

- Yes, I have access to echocardiographic devices and use them when/if needed
- Yes, I have access to echocardiographic devices, but cannot use them as often as needed
- Yes, I have access to echocardiographic devices, but I don't use them because of lack of training/expertise
- No, echocardiographic devices are not available where I work
- Other :

**11. MULTIPLE ANSWERS POSSIBLE: Which ultrasound DEVICES do you have access to?**

- Classic ultrasound machine on a trolley/cart
- Miniaturized pocket ultrasound device
- I don't have access to any ultrasound devices

**12. MULTIPLE ANSWERS POSSIBLE: BEFORE administering a FLUID BOLUS, do you try to predict fluid RESPONSIVENESS?**

- Yes, I use Pulse Pressure Variation (PPV) when possible
- Yes, I use Stroke Volume Variation (SVV) when possible
- Yes, I perform a Passive Leg Raising (PLR) test when possible
- Yes, I perform another test (tidal volume challenge, end-expiratory occlusion test...)
- Yes, I use ULTRASOUND indices to do so
- No, I do NOT try to predict fluid responsiveness BEFORE administering a fluid bolus
- Other :

**13. MULTIPLE ANSWERS POSSIBLE: To assess tissue PERFUSION**

- I rely on clinical evaluation (skin temperature, mottling, urine output…)
- I quantify the Capillary Refill Time (CRT)
- I measure blood lactate
- I monitor the peripheral perfusion index with a pulse oximeter
- I use a hand-held video microscope to visualize the sublingual microcirculation
- I do NOT assess tissue perfusion
- Other :

**14. To assess tissue OXYGENATION, do you have access to NIRS (Near InfraRed Spectroscopic) sensors to assess brain and/or muscle oxygenation?**

- Yes, and I use them when/if needed
- Yes, but I don't use them as often as needed
- Yes, but I don't use them because of lack of training/expertise
- No, they are not available where I work
- Other :

**15. FINAL QUESTION: Do you give us permission to use your answers for ANONYMOUS analysis and related publication?**

- Yes
- No
